# Supplementary material for: Pollen as Bee Medicine: Is Prevention Better than Cure?
Source: Biology (Basel). 2023 Mar 24;12(4):497. doi: 10.3390/biology12040497 (PMC10135463; doi:10.3390/biology12040497)
Supplement: Supplementary file 1 [file biology-12-00497-s001.zip › biology-2208199-supplementary.pdf]

Table 1. Prophylactic and therapeutic effects of different pollen diets. Parameters related to host tolerance and host resistance are presented (mean±SD). Data worker fat body, worker mortality and larval ejection are expressed as proportion (no unit). For host resistance, the dynamics of parasite load over time is presented.

	Parameters	Uninfected control	Prophylactic treatments				
			Multifloral	Willow	Sunflower	Poppy	Heather
Tolerance	Brood mass (g)	2.59±0.99	3.52±0.77	2.52±1.38	0.68±0.61	2.05±1.26	2.16±1.20
	Worker fat body	0.15±0.03	0.12±0.02	0.13±0.03	0.12±0.02	0.16±0.11	0.12±0.03
	Worker mortality	0.06±0.09	0.10±0.16	0.17±0.15	0.05±0.10	0.05±0.10	0.02±0.06
	Larval ejection	0.01±0.01	0.02±0.02	0.02±0.04	0.19±0.27	0.00±0.00	0.08±0.11
	Pollen efficiency (g/g)	0.75±0.16	0.87±0.09	0.76±0.21	0.25±0.22	0.68±0.29	0.67±0.34
Resistance	Parasite (cells.μL^{-1}, inoc. at day 1)						
	Day 4	NA	622±349	1456±801	500±362	4317±4521	1078±1079
	Day 7	NA	19828±11121	11142±7210	712±677	15306±13523	1478±1335
	Day 10	NA	46861±69868	28333±17310	11806±17628	21306±15645	23167±30530
	Day 13	NA	214750±173708	94361±96319	24306±19780	80472±66032	33056±39889
	Parasite growth (index)	NA	562±691	123±180	97±109	37±43	87±116
		**Uninfected control**	**Therapeutic treatments**				
			**Multifloral**	**Willow**	**Sunflower**	**Poppy**	**Heather**
Tolerance	Brood mass (g)	2.59±0.99	1.80±1.33	1.90±1.29	0.37±0.34	1.26±1.09	1.24±0.84
	Worker fat body	0.15±0.03	0.15±0.03	0.15±0.04	0.14±0.02	0.14±0.03	0.11±0.03
	Worker mortality	0.06±0.09	0.10±0.10	0.04±0.07	0.06±0.08	0.02±0.06	0.07±0.15
	Larval ejection	0.01±0.01	0.02±0.03	0.02±0.04	0.18±0.12	0.00±0.00	0.04±0.03
	Pollen efficiency (g/g)	0.75±0.16	0.57±0.35	0.67±0.29	0.14±0.11	0.54±0.39	0.49±0.25
Resistance	Parasite (cells.μL^{-1}, inoc. at day 1)						
	Day 4	NA	328±256	207±116	39±70	1200±1067	150±287
	Day 7	NA	23039±46484	6706±14627	1833±4107	7000±3531	8769±16465
	Day 10	NA	51083±80244	21283±16293	1433±933	10111±5765	3733±3852
	Day 13	NA	33129±19166	32917±31533	19722±12151	21333±12109	27471±27683
	Parasite growth (index)	NA	212±215	167±99	328±261	51±50	354±350

Table S2. Pollen collection in microcolonies offered a choice between two pollen diets. The daily pollen collection over time is presented, as well as the total pollen collection at the end of the bioassay.

Parameters	Multifloral *vs* Sunflower		Multifloral *vs* Heather		Sunflower *vs* Heather	
	Multifloral	Sunflower	Multifloral	Heather	Sunflower	Heather
			Infected			
Daily pollen collection (g)						
Day 6	0.068±0.032	0.044±0.027	0.085±0.032	0.024±0.044	0.036±0.032	0.027±0.028
Day 7	0.066±0.045	0.036±0.037	0.091±0.020	0.008±0.011	0.063±0.041	0.021±0.022
Day 8	0.051±0.046	0.043±0.035	0.045±0.023	0.019±0.028	0.031±0.025	0.043±0.031
Day 9	0.077±0.038	0.006±0.009	0.099±0.043	0.007±0.010	0.086±0.047	0.023±0.040
Day 10	0.030±0.022	0.062±0.039	0.060±0.027	0.035±0.039	0.050±0.041	0.052±0.040
Day 11	0.067±0.030	0.026±0.019	0.073±0.031	0.018±0.016	0.073±0.043	0.025±0.022
Day 12	0.065±0.052	0.069±0.056	0.078±0.033	0.050±0.076	0.049±0.063	0.086±0.060
Total pollen collection (g)	0.426±0.143	0.287±0.104	0.528±0.095	0.162±0.172	0.384±0.164	0.277±0.160
			Uninfected			
Daily pollen collection (g)						
Day 6	0.091±0.038	0.064±0.056	0.087±0.048	0.036±0.033	0.033±0.045	0.040±0.038
Day 7	0.101±0.021	0.024±0.036	0.071±0.020	0.019±0.028	0.073±0.031	0.014±0.013

Day 8	0.020±0.015	0.054±0.033	0.032±0.027	0.034±0.019	0.039±0.031	0.036±0.024
Day 9	0.121±0.031	0.028±0.047	0.108±0.021	0.019±0.039	0.080±0.018	0.017±0.022
Day 10	0.038±0.013	0.087±0.038	0.059±0.037	0.036±0.024	0.045±0.029	0.047±0.043
Day 11	0.091±0.036	0.032±0.036	0.062±0.039	0.018±0.012	0.081±0.025	0.022±0.017
Day 12	0.062±0.041	0.087±0.035	0.122±0.104	0.059±0.049	0.047±0.038	0.070±0.067
Total pollen collection (g)	0.526±0.078	0.376±0.195	0.540±0.151	0.222±0.127	0.398±0.140	0.244±0.159
